# Attentional biases toward body images in males at high risk of muscle dysmorphia

**DOI:** 10.7717/peerj.4273

**Published:** 2018-01-16

**Authors:** Xinhong Jin, Yahong Jin, Shi Zhou, Shun-nan Yang, Shuzhi Chang, Hui Li

**Affiliations:** 1 General Sport Administration of China’s Key Laboratory of Sports Psychological and Physiological Regulation, School of Education and Psychology, Tianjin University of Sport, Tianjin, People’s Republic of China; 2 Department of Sport Psychology, School of Kinesiology, Shanghai University of Sport, Shanghai, People’s Republic of China; 3 School of Health and Human Sciences, Southern Cross University, Lismore, NSW, Australia; 4 Vision Performance Institute, College of Optometry, Pacific University, Forest Grove, OR, USA

**Keywords:** Muscle dysmorphia, Attentional bias, Eye movements, Self-schema, Body image

## Abstract

**Objective:**

Although research on muscle dysmorphia (MD), a body dysmorphic disorder subtype, has recently increased, the causes and mechanisms underlying this disorder remain unclear. Results from studies examining disorders associated with body image suggest the involvement of self-schema in biasing attention toward specific body information. The present study examined whether individuals at higher risk of MD also display attentional biases toward specific types of body images.

**Methods:**

The validated Chinese version of the Muscle Appearance Satisfaction Scale was used to distinguish men at higher and lower risk of MD. Sixty-five adult Chinese men at higher (HRMD, *n* = 33) and lower risk of MD (LRMD, *n* = 32) performed a visual probe task. Initially, an image of a bodybuilder with either larger or smaller musculature was presented on one side of a central point, with a neutral image of a car exterior presented on the other side along the horizontal plane for 2,000 ms. The paired images were removed, and a visual target (a dot) was displayed in the location of one of the previously shown images. Participants were asked to indicate the location of the target, and their eye movements were recorded during the entire visual presentation. Participant reaction time and three eye movement measurements (gaze direction, first saccade latency, and first fixation duration) were recorded for use in determining attentional bias.

**Results:**

The HRMD group revealed biases in orienting and maintaining their attention on images of bodybuilders with larger musculatures. Participants in this group consequently had a shorter reaction time in identifying the target that appeared at the location in which an image of a bodybuilder with a larger musculature had been previously displayed. They also directed their initial gaze more frequently, had shorter saccade latency, and had longer first fixation duration on images of bodybuilders with larger musculatures (all *p* < .0001). In comparison, the LRMD group had longer reaction times, slower attention orientation toward body images, and shorter fixation duration for images of bodybuilders with larger musculatures (all *p* < .0001), indicating weaker or mixed responses.

**Discussion:**

Adult Chinese men at higher risk of MD displayed biases in orienting and maintaining their visual attention toward images of bodybuilders with larger musculatures, and these biases facilitated their information processing. These results suggest that development of MD may be due in part to attentional biases associated with established negative self-schema of specific body information. These findings provide insight into understanding and identifying the cognitive characteristics of MD in an Asian population.

## Introduction

Visual perception is affected by not only image saliency but also personal knowledge biases (Cho & Lee, 2012; Gregory, 1997). The perception and subsequent emotional evaluation of one’s own body is similarly mediated by a set of personal beliefs about body image. In extreme cases, such beliefs may be distorted and lead to recognized mental disorders, such as body image disorders and eating disorder (ED) (Cordes et al., 2017; Gao et al., 2011; Griffiths et al., 2014). However, the mechanisms underlying the development of these disorders are not well understood.

Recently, there has been an increase in research in both Western and non-Western cultures focused on muscle dysmorphia (MD), a subtype of body dysmorphic disorder that predominantly affects males (American Psychiatric Association, 2013). Men with MD believe that they are insufficiently muscular. They are often preoccupied with thoughts that they look puny or small despite their normal or even muscular appearance, and they are usually ashamed of their appearance (Olivardia, 2001). An increasing prevalence of MD has also been reported in the mass media, such as the British Broadcasting Corporation, which reported that 10% of male gym members in the United Kingdom experienced MD (Ahmad, Rotherham & Talwar, 2015). People with MD spend much of their time involved in muscle enhancement activities, adding the possibilities of exercise addiction, drug addiction, or impaired quality of life (Tod, Edwards & Cranswick, 2016).

These distorted beliefs about one’s own body might be best attributed to the existence of a negative “self-schema.” According to Markus (1977), a self-schema is the cognitive generalization about oneself that helps organize and guide the processing of self-related information. Once established, the self-schema acts as a biasing mechanism to influence the selection, organization, and evaluation of the information. When individuals form a negative schema of their own body image, they may direct their attention toward the processing of body information consistent with this negative generalization.

A handful of researchers have studied information processing bias for women who typically desire thinness (Cho & Lee, 2012; Gao et al., 2011; Joseph et al., 2016). Individuals with ED showed a decreased likelihood of visually fixating on their own “beautiful” body features and an increased frequency of fixating on “ugly” body features (Jansen, Nederkoorn & Mulkens, 2005). Biases are also found in negative and neutral shape stimuli (Shafran et al., 2007). This empirical evidence suggests that self-schema plays a role in focusing attention on specific aspects of visual information.

Body dissatisfaction has also been studied in men who aim to be stronger, with larger muscles and a well-toned body (Cordes et al., 2016; Cordes et al., 2017; Joseph et al., 2016). Individuals with body dissatisfaction show attentional bias toward images of bodies when countering information from their own bodies or others (Cho & Lee, 2012; Cordes et al., 2016; Cordes et al., 2017; Joseph et al., 2016), specific stature dissatisfaction (Liu et al., 2014), and emotion regulation difficulties (Griffiths et al., 2014). Although these findings reveal an increased focus on specific body information, these studies did not examine the mechanisms underpinning such an attentional bias. A similar study conducted in weight-dissatisfied women found biases in both orienting and maintaining visual attention on words related to fatness rather than on neutral words (Gao et al., 2011). However, the use of abstract words instead of visual images in that study may have selectively engaged top-down attentional processes and artificially exaggerated the influence of self-schema on information selection.

Recent research has provided converging evidence on an underlying association between developing MD and body image disorders (Phillipou, Blomeley & Castle, 2015). People with MD may expend considerable time and energy trying to become more muscular and to avoid social or vocational activities because of their shame over their perceived negative body image (Pope et al., 2005). However, such actions contribute to additional mental disturbances or psychiatric comorbidities related to body image (Behar & Molinari, 2010; Cafri, Olivardia & Thompson, 2008).

The results of several studies investigating the male drive for muscularity and those examining ED and body dissatisfaction displayed marked similarities in how visual perception is biased toward specific body information (Cho & Lee, 2012; Cordes et al., 2016; Griffiths et al., 2014; Phillipou, Blomeley & Castle, 2015). These results have also suggested the involvement of negative self-schema in biasing one’s attention toward body information. Recent studies, some using eye-tracking methodologies to examine body-directed visual attention, are increasingly focused on body dissatisfaction among males (Cordes et al., 2016; Cordes et al., 2017; Griffiths et al., 2014; Joseph et al., 2016). The pursuit of muscularity in Chinese culture remains mostly associated with the male population (Tod, Edwards & Cranswick, 2016). However, attentional bias toward body information among individuals who are at high risk of MD (HRMD) has not yet been examined. Thus, the present study investigated whether the attentional bias shown by populations with ED and body dissatisfaction is also found in Chinese males with HRMD.

According to Duc, Bays & Husain (2008), eye movement, which can determine where and for how long attention has been directed, plays a vital role in cognitive-attentional processes. Indeed, examining dwell time and allocation using eye-tracking methodology has been shown to be a suitable approach in investigating attentional bias (Cordes et al., 2016; Cordes et al., 2017; Gao et al., 2011). In addition, visual dot-probe paradigms have been used to assess whether attention is directed toward or away from particular types of stimuli and subsequent targets (Gao et al., 2011), taking advantage of examining attentional bias for cognitive processing and reaction time to the onset of a subsequent target. A visual probe task similar to that used by Gao et al. (2011) was adopted in the present study. Thus, the present study combined reaction time assessment and eye-movement tracking to provide insight into the mechanisms underlying attentional bias in a population of Chinese men with HRMD. Based on the results of previous studies examining attentional bias in individuals with body dissatisfaction and in those with ED, we hypothesized that men in the HRMD group would show an attentional bias toward images of bodybuilders with a large musculature and would persist in maintaining their attention on the location in which these images had been shown.

## Methods

### Participants

#### Participant recruitment

Two hundred participants were recruited from 10 fitness centers and gyms in Tianjin, China. All recruited participants worked out with free weights or machines at least three times per week. They answered a Chinese version of the Muscle Appearance Satisfaction Scale (CMASS; Jin et al., 2015) questionnaire. Their total scores on the CMASS were ranked in descending order. Participants scoring in the top 27% (*n* = 54, total scores 51–72) comprised the HRMD group, as they were considered to be at the highest risk of developing MD, whereas participants scoring in the bottom 27% (*n* = 54, total scores 24–42) comprised the low risk of MD (LRMD) group (Kelley, 1939).

#### Eye-tracking experiment participants

On the basis of the assumed effect size and correlation calculated from a previous study (Jin et al., 2015), 33 participants were recruited from each of the HRMD and LRMD groups for inclusion in the eye-tracking experiment to achieve a statistical power of 80% (see File S1 for more details about the recruiting procedure). One participant did not finish the study, resulting in a total of 65 participants, who ranged in age from 20 to 33 years (mean, 23.66 years; SD, 2.08 years). Similar to previous eye-tracking studies (Castellanos et al., 2009; Gao et al., 2011), all participants in the present study were right-handed, had no prior experience with a similar experiment, had no color blindness or other eye disease, and had normal or corrected-to-normal vision.

### CMASS and demographic questionnaires

#### Demographics

Demographic questions asked though a self-reported questionnaire included participant age, handedness, vision, history of weight training (years, frequency, and time for each training), history of color blindness or other eye disease, and history of neurological or psychiatric illness. History of eating disorders and cosmetic surgery were also surveyed.

#### Chinese version of muscle appearance satisfaction scale

The CMASS is a 17-item, self-reported questionnaire, with total scores ranging from 17 to 85. Higher scores on the MASS reflect a tendency toward MD (Babusa et al., 2012; González-Martí et al., 2012; Mayville et al., 2002; Ryan & Morrison, 2010). The MASS has been adapted to different languages and has been shown effective for assessing symptoms of MD (Babusa et al., 2012; González-Martí et al., 2012). The CMASS, which includes five subscales (muscle checking, muscle satisfaction, substance use, injury, and bodybuilding dependence; Jin et al., 2015) has been shown to have good construct validity (CFI, 0.931; RMSEA, 0.052) and adequate internal consistency for all five factors (Cronbach *α*, .636 to .737) in adult Chinese men.

### Eye-tracking apparatus

Eye movements were recorded with a head-mounted Eyelink II eye tracker (SR Research, Osgoode, Ontario, Canada). The eye tracker had a sample rate of 250 Hz and a spatial accuracy of 0.1°. An integrated infrared head camera was used to measure head motion and provide corrected eye positions. A forehead/chin rest was used to keep the viewing distance constant and to minimize head movements. The distance between participants’ eyes and the screen was approximately 65 cm.

Test stimuli were presented on a 19″ LCD screen with a resolution of 1,024 × 768 pixels and a vertical refresh rate of 60 Hz. The center of the screen was aligned to the location between participant’s two eyes and was perpendicular to the line of eyesight.

### Pictures viewed

Test images were prepared based on recommendations by Gao et al. (2011) and included images of car exteriors (neutral) and bodybuilders with large or small musculatures. Car images were taken with permission from websites, and bodybuilder images were obtained with permission from websites or captured by professional photographers employed by the authors. The body images showed seven poses used to display the muscles typically developed for bodybuilding competitions (front double biceps, front lateral spread, side chest, side triceps, rear double biceps, rear lateral spread, and abdominal and thigh poses).

Before the eye-tracking experiment, a pilot study was conducted to assess and screen the aforementioned images. For that pilot study, 50 male undergraduate students who did not participate in the formal eye-tracking experiment were recruited. The arousal and valence of each car image was evaluated by the students using the self-assessment manikin proposed by Bradley & Lang (1994), and the musculature of each close-up image was assessed using a 9-point Likert scale (more details for File S2). On the basis of these student assessments, the body images with the top 10 total scores were categorized as bodybuilders with large musculature for use in the formal eye-tracking experiment, and those with the bottom 10 total scores were considered bodybuilders with small musculature. The 40 car images with middle scores in perceived arousal and valence were used as neutral images in the formal eye-tracking experiment. An additional 30 images of cars with middle scores in perceived arousal and valence were used as practice materials for the experiment.

All images used in the experiment were composed of a black background and a single foreground image 8.2 cm in width and 9.6 cm in height (visual angle was 8.46°). The images were displayed on a computer screen as part of the visual probe task using E-prime 2.0 software (Psychology Software Tools Inc., Pittsburgh, PA, USA).

### Procedure

Ethical approval for this study was obtained from the Tianjin University of Sport Research Ethics Committee. Participants read and signed an informed consent form in accordance with the principles specified in the Declaration of Helsinki. After giving consent, they provided their demographic information, types and frequencies of free-weight exercises, and contact information and completed the CMASS questionnaire.

To evaluate any bias in participant reaction time, measurements of simple reaction time for each participant were conducted with a modified version of the visual probe task in which the central fixation point was switched off after 1,000 ms and the dot probe was presented at either the right or left sides of the fixation cross. The participant was asked to press the “A” key as soon as they detected the probe. In total, 20 trials were conducted to obtain an average reaction time. All participants then underwent the attentional bias-related eye-tracking experiment.

At the beginning of the eye-tracking experimental session, participants sat directly in front of the computer display, and the chair height was adjusted to allow comfortable use of the forehead/chin rest. They first calibrated their gaze position with a nine-point calibration pattern; practice and formal trials were conducted after satisfactory calibration.

The present study used the visual probe task modified from an earlier study (MacLeod & Mathews, 1988). Each trial began with the display of a central fixation cross for 1,000 ms, followed by a pair of test images shown at the right and left sides of the fixation cross for 2,000 ms. The distance between the centers of the paired images and the fixation cross was 5 cm. There were three types of image pairs (bodybuilder with large musculature-neutral, bodybuilder with small musculature-neutral, and neutral-neutral), with the location of paired images randomly assigned across trials. Neutral-neutral pairs were fillers aimed to conceal the purpose of the experiment (Castellanos et al., 2009). After the images were presented, a dot was shown at the center of one of the now-removed images. Participants were told that their main task was to indicate the position of the dots as soon and as accurately as possible. They were to press the “A” key when the dot was presented at the left location and press the “L” key when the dot was presented at the right location. The dot was removed and the trial terminated when a key response was detected, or when no response was given after 5,000 ms. The next trial was commenced immediately after the termination of the preceding trial (File S3). There were 15 practice trials that used car images excluded from the formal trials. Four blocks of formal trials were conducted after practice trials, with 30 trials in each block and 120 total trials. Breaks were scheduled between trial blocks, and calibration was conducted after each break.

### Statistical analysis

#### Reaction time

Data from practice and filler trials were discarded. Reaction times less than 200 ms or two standard deviations above the mean were also excluded from analysis (Castellanos et al., 2009; Gao et al., 2011). For the remaining data, the error rates of key responses for each participant were between 0.83% and 2.5%. A missed response in a trial was encoded as an error response. Only the reaction time of accurate responses was included for further analysis.

Participant “reaction time bias” was calculated based on the equation [(*B*_*l*_*D*_*r*_ − *B*_*r*_*D*_*r*_) + (*B*_*r*_*D*_*l*_ − *B*_*l*_*D*_*l*_)]∕2, where the four mean reaction times were calculated based on the type and location of image and probe (B indicates bodybuilder image, *D*, dot probe; *l*, left; and *r*, right; MacLeod & Mathews, 1988). A positive reaction time bias reflects a tendency to attend to body images, whereas a negative reaction time bias reflects a tendency to avoid body images.

#### Eye movement data

Eye movement outcomes were included in the analysis only when the following criteria were satisfied: (a) During the initial fixation period, the gaze was maintained within a spatial window of 1° of visual angle from the center of fixation cross for at least 100 ms; (b) the gaze was within the same spatial window when the pair of images appeared; (c) the initial saccade outside of the spatial window occurred at least 100 ms after the onset of paired images; and (d) the initial fixation outside of the spatial window was on either the left or right image rather than the empty space (Castellanos et al., 2009; Gao et al., 2011). Outcomes from trials not meeting these criteria (8.12%) as well as those from the practice and filler trials were excluded from further analysis.

Three measurements of attentional biases were derived from eye movement data, as suggested in previous studies (Castellanos et al., 2009; Gao et al., 2011): gaze direction, first saccade latency, and first fixation duration. The gaze direction bias was obtained by computing the percentage of trials in which the body image was fixated on first. A gaze direction bias greater than 0.5 suggests an orienting bias toward body images. The bias for the first saccade latency was obtained by deducting the latency for the first saccade on the neutral image from that on the body image for trials, with results for large and small musculature images computed separately. Here, a negative score reflects faster orientation toward a body image, whereas a positive score reflects slower orientation. The bias for the first fixation duration was calculated by subtracting the first fixation duration for the neutral image from that for the body image, again computed separately for body images with large or small musculature. A score greater than 0 reflects maintained attention toward a body image, whereas a score less than 0 reflects an initial avoidance of a body image (Castellanos et al., 2009; Gao et al., 2011). The biases for gaze direction and first saccade latency represent orienting biases, whereas that for the first fixation duration indicates a maintenance bias.

All raw data were collected and entered into statistical analysis programs using SPSS version 19.0 software (IBM, Chicago, IL, USA). Mixed-model analysis of variance (ANOVA) was conducted to assess the effect of participant group (HRMD vs. LRMD, between subject) and image type (large vs. small musculature, within subject) on three eye-movement indices (gaze direction bias, first saccade latency bias, and first fixation duration bias) as well as the reaction time bias. If there were interaction effects, analyses of the simple effects were conducted. The homogeneity of data distribution was tested, and a significant violation of homogeneity was mitigated by correcting the resultant degree of freedom and *p* value based on the procedure suggested by Greenhouse & Geisser (1959). An *α* value of .05 was used for determining the statistical significance for all analyses.

## Results

### Sampling validity

The frequency of exercise as well as the total and subscale CMASS scores were calculated for both the HRMD and LRMD groups to assess whether the risk of MD was distinguished in the groups (Table 1). The results of independent *t*-tests showed a significant difference between the groups (*p* <   .0001) for the total and subscale CMASS scores but not for the frequency of exercise, indicating that the HRMD group had more typical characteristics and risk of MD than did the LRMD group.

**Table 1:** Comparison with CMASS scores and frequency of excercise between the HRMD and LRMD groups.

	Group	*N*	Score (*M ± SD*)	*t*
Muscle Checking	HRMD	33	11.48 ± 2.72	4.50^***^
	LRMD	32	8.75 ± 2.14	
Muscle Satisfaction	HRMD	33	9.73 ± 1.96	.97
	LRMD	32	9.22 ± 2.25	
Substance Use	HRMD	33	7.12 ± 2.32	3.89^***^
	LRMD	32	5.16 ± 1.71	
Injury	HRMD	33	10.09 ± 2.11	5.22^***^
	LRMD	32	7.16 ± 2.41	
Bodybuilding Dependence	HRMD	33	12.30 ± 2.72	4.25^***^
	LRMD	32	9.41 ± 2.78	
Total Score	HRMD	33	50.73 ± 6.51	7.07^***^
	LRMD	32	39.69 ± 6.07	
Frequency of Exercise	HRMD	33	3.97 ± 1.92	1.40
	LRMD	32	3.40 ± 1.10	

**Notes.**

CMASS the Chinese version of the Muscle Appearance Satisfaction Scale HRMD Higher Risk of Muscle Dysmorphia LRMD Lower Risk of Muscle Dysmorphia

*** *p* <.0001.

### Reaction time bias

We evaluated the simple reaction time of participants in the two groups. The results of independent *t*-tests showed no significant difference between the HRMD and LRMD groups (*t*_63_ =  − .357; *p* = .722; Cohen’s *d* =  − .088). This indicated that the calculated reaction time bias was not affected by the speed of the manual response between the two groups.

The results of a mixed-model ANOVA based on reaction time bias revealed no main effect of image type (*F*_(1,63)_ = 1.43 *p* = .237, ${\eta }_{p}^{2}=.022$; Table 2); however, there was a significant main effect of participant group (*F*_(1,63)_ = 9.35 *p* = .003, ${\eta }_{p}^{2}=.129$) and a significant interaction between participant group and image type (*F*_(1,63)_ = 12.97, *p* = .001, ${\eta }_{p}^{2}=.171$). Analyses of simple effects indicated that for images of bodybuilders with large musculatures, individuals in the HRMD group showed a positive reaction time bias, whereas those in the LRMD group had a negative bias (*F*_(1,63)_ = 45.71, *p* < .0001, ${\eta }_{p}^{2}=.420$), indicating a significant tendency to attend to and to avoid muscular body images in the HRMD and LRMD groups, respectively. In addition, the reaction time in the LRMD group was markedly and significantly affected by image type, as the reaction time bias differed in this group based on the muscle size in the images, with a positive reaction time bias for images with smaller musculature and a negative reaction time bias for images with large musculature (*F*_(1,63)_ = 11.32, *p* = .001, ${\eta }_{p}^{2}=.152$).

**Table 2:** Reaction time and computed bias for the HRMD and LRMD groups (ms, M ±  SE).

Location		Reaction time and bias (*M ± SE*)	
Body image	Probe	HRMD group *N* = 33	LRMD group *N* = 32
Large vs. Neutral			
Left	Right	447.62 ± 5.98	419.66 ± 5.36
Left	Left	446.10 ± 7.94	435.27 ± 7.34
Right	Left	461.28 ± 7.02	426.83 ± 5.65
Right	Right	432.98 ± 5.99	428.96 ± 6.01
Bias		22.78 ± 4.19	−9.54 ± 5.82
Small vs. Neutral			
Left	Right	452.34 ± 7.28	424.54 ± 6.88
Left	Left	445.27 ± 6.48	416.74 ± 5.10
Right	Left	461.40 ± 8.66	439.79 ± 8.44
Right	Right	446.61 ± 6.60	420.70 ± 5.22
Bias		12.65 ± 5.62	16.43 ± 9.58
Variance			*F*
Image type			1.43
Subject group			9.35^**^
Image type × subject group			12.97^**^

**Notes.**

HRMD Higher Risk of Muscle Dysmorphia LRMD Lower Risk of Muscle Dysmorphia

** *p* <.01.

**Figure 1:**
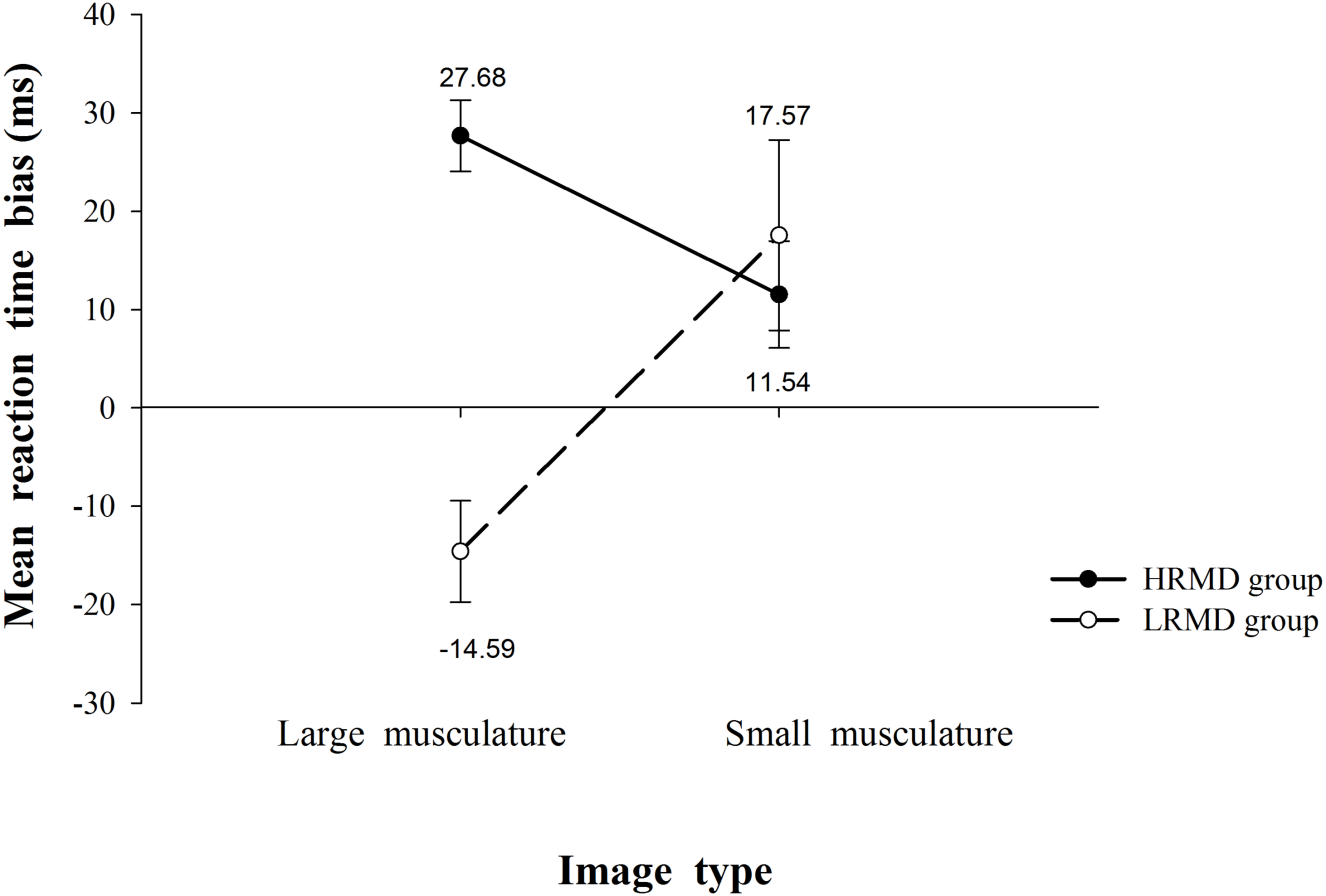
Mean reaction time bias associated with participant group and image type. A positive number indicates a faster response by the participants in that group for an image with the indicated body type (small vs. large musculature). Error bars indicate 95% confidence intervals.

To further explore the significance of the observed reaction time bias, one-sample *t*-tests were conducted to assess the reaction time bias relative to zero in both groups. Figure 1 shows that for the HRMD group the reaction time bias was significantly greater than zero (i.e., reaction time for body image < for neutral image) for images with large musculature (*t*_32_ = 7.67, *p* <   .0001) but only marginally significant for those with smaller musculature (*t*_32_ = 2.13, *p* = .041), suggesting a positive reaction bias toward body images with larger musculature in this group. For the LRMD group, the reaction time bias was significantly less than zero for images with larger musculature (i.e., reaction time for body image >  for neutral image; *t*_31_ =  − 2.84, *p* = .008) but was not significantly different from zero for images with smaller musculature (*t*_31_ = 1.82, *p* = .079), indicating a negative reaction bias toward body images with larger musculature.

Taken together, these results indicated that both the HRMD and LRMD groups have an attentional bias toward body images with large muscles. However, whereas the HRMD group showed a positive bias, indicating a tendency to attend to body images with large muscles, the LRMD group had a negative bias, reflecting an avoidance of body images with large muscles.

### Gaze direction bias

**Figure 2:**
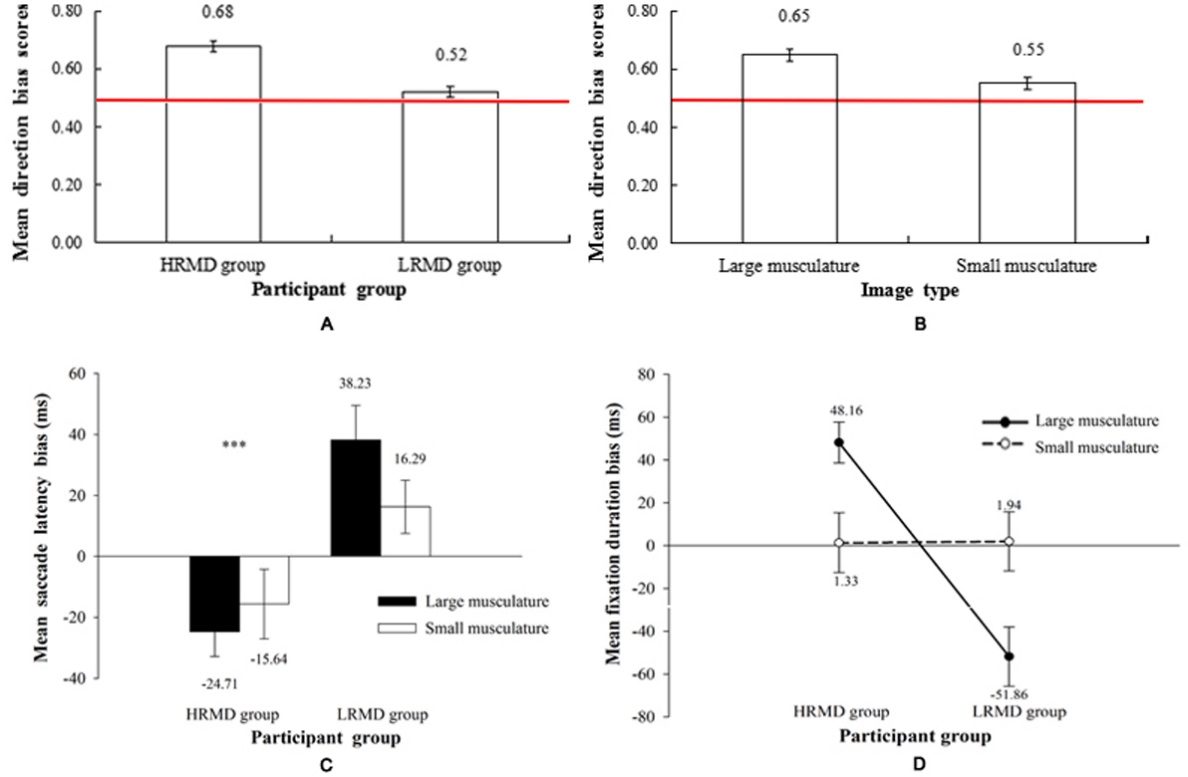
Eye movement data for participant group and image type. (A) Mean gaze direction bias relative to participant group. Error bars indicate 95% intervals. A value above 0.5 (red line) indicates a higher probability of the gaze orientated toward the body image (^∗∗∗^*p* < .0001). (B) Mean gaze direction bias relative to image type in the HRMD group. Error bars indicate 95% intervals. A value above 0.5 (red line) indicates a higher probability of the gaze orientated toward the body image (^∗∗∗^*p* < .0001). (C) Mean saccade latency bias relative to participant group and image type. Error bars indicate 95% confidence intervals (^∗∗∗^*p* < .0001). (D) Mean fixation duration bias relative to the participant group and image type. A positive value indicates a longer duration for the initial fixation on the body image. Error bars indicate 95% confidence intervals.

The results of a mixed-model ANOVA for initial gaze direction bias revealed significant main effects of participant group (*F*_(1,63)_ = 23.34, *p* <  .0001, ${\eta }_{p}^{2}=.270$) and image type (*F*_(1,63)_ = 42.03 *p* <.0001, ${\eta }_{p}^{2}=.40$). The gaze direction bias for the HRMD group was significantly greater than that for the LRMD group (*t*_128_ = 5.90, *p* <  .0001, Cohen’s *d* = 1.037; Fig. 2A), which indicated more attention capture for the HRMD group. In addition, a greater bias was found in the HRMD group for images with larger musculature than for those with smaller musculature (*t*_128_ = 3.40, *p* = .001, Cohen’s *d* = 0.597; Fig. 2B), suggesting increased attention to images of large muscles in this group of males. There was no interaction between participant group and image type (*F*_(1,63)_ = 0.66, *p* = .420, ${\eta }_{p}^{2}=.01$).

One-sample *t*-tests revealed that the HRMD group had a gaze direction bias (greater than 0.5) for images of bodybuilders with both large (*t*_32_ = 9.59, *p* <   .0001) and small muscles (*t*_32_ = 4.36, *p* <  .0001). For the LRMD group, a gaze direction bias was found for images of bodybuilders with large muscles (*t*_31_ = 2.57, *p* = .015) but not with small muscles (*t*_31_ =  − 0.91, *p* = .370).

These results indicated a gaze direction bias toward images of bodybuilders with larger muscles among all participants. Furthermore, the HRMD group had significant attention capture for body images with either large or small muscles, whereas the LRMD group had attention capture only for images with large muscles.

### First saccade latency bias

The results of a mixed-model ANOVA revealed a significant main effect for participant group (*F*_(1,63)_ = 16.62 *p* <.0001, ${\eta }_{p}^{2}=.209$). There was a larger negative latency bias for the first saccade in the HRMD group than in the LRMD group (*t*_128_ =  − 4.74, *p* <  .0001, Cohen’s *d* =  − 0.830). The LRMD group had a larger positive latency bias, indicating a slower orientation toward the body image, whereas individuals in the HRMD group showed a smaller negative latency bias, suggesting a faster orientation toward the body images. No effect was observed for image type (*F*_(1,63)_ = 0.65, *p* = .424, ${\eta }_{p}^{2}=.01$) or for the interaction between participant group and image type (*F*_(1,63)_ = 3.76, *p* = .057, ${\eta }_{p}^{2}=.056$).

As shown in Fig. 2C, one-sample *t*-tests further revealed that for the LRMD group the bias in first saccade latency was marginally significantly greater than zero for images of bodybuilders with large muscles (*t* = 2.12, *p* = .042) but not for those with small muscles (*t* = 1.75, *p* = .089).

These results indicated that the LRMD group showed slower attention orientation toward body images, whereas the HRMD group oriented faster toward body images.

### First fixation duration bias

The results of a mixed-model ANOVA for first fixation duration bias demonstrated a significant main effect for participant group (*F*_(1,63)_ = 12.10 *p* = .001, *η* ${}_{p}^{2}=.161$) but not for image type (*F*_(1,63)_ = 0.58 *p* = .449, ${\eta }_{\mathrm{p}}^{2}=.009$). However, the interaction between group and image type was significant (*F*_(1,63)_ = 20.54 *p* <.0001, ${\eta }_{\mathrm{p}}^{2}=.246$). Analyses of simple effects showed that the HRMD group had a positive bias in the first fixation duration on the images with large muscles, whereas the LRMD group showed a negative bias (*F*_(1,63)_ = 32.79 *p* <  .0001, ${\eta }_{\mathrm{p}}^{2}=.342$). Participants in the HRMD group were more likely to show a positive fixation duration bias for images with large rather than small muscles (*F*_(1,63)_ = 7.22, *p* = .009, ${\eta }_{\mathrm{p}}^{2}=.103$), indicating their attention was maintained longer on the images with large muscles. By contrast, participants in the LRMD group showed a negative fixation duration bias for images with large muscles (*F*_(1,63)_ = 13.80, *p* <  .0001, ${\eta }_{\mathrm{p}}^{2}=.180$; Fig. 2D), indicating a longer attention avoidance of such images.

One-sample *t*-tests revealed that for images of bodybuilders with large muscles, the direction of the fixation duration bias scores differed between the HRMD and LRMD groups such that they were significantly greater than zero in the HRMD group (*t*_32_ = 5.07, *p* <  .0001) but significantly less than zero in the LRMD group (*t*_31_ =  − 3.64, *p* = .001), indicating a maintained attention of HRMD individuals for body images but an initial avoidance by the LRMD individuals.

Taken together, these results indicated maintained attention on images of bodybuilders with large muscles in the HRMD group but an initial attention avoidance of these images in the LRMD group. In addition, individuals in the HRMD group maintained attention longer on images of bodybuilders with larger rather than smaller muscles.

## Discussion

The present study revealed that adult Chinese males with HRMD displayed attentional biases toward images of bodies. During a visual probe task, their reaction time toward the location of an image of a body was faster than that toward a neutral image (a car exterior). Compared with the measures for the neutral image, participants in the HRMD group also had a higher likelihood of visually orienting toward the images of a body, took less time to shift their gaze toward it, and maintained fixation on it longer. Those with LRMD showed weaker and mixed tendencies of doing so. Furthermore, for men with HRMD, compared with the results from images of bodybuilders with smaller musculatures, images of bodybuilders with large musculatures were associated with a shorter reaction time, shorter saccade latency, and longer fixation duration. A significant interaction was found between reaction time and first fixation duration; that is, the HRMD group was both quicker to attend to and spent more time initially looking at images of the more muscular bodybuilders than the LRMD group did. Together, these outcomes suggest clear biases in both directing and maintaining visual attention on images of large musculature for men with HRMD. Those with LRMD showed no such attentional biases.

Previous studies on ED have shown an attentional bias toward unfavorable body images (Jansen, Nederkoorn & Mulkens, 2005) and the guidance and maintenance of visual attention on negative body-related words (Gao et al., 2011). The present study separately measured the orientation and maintenance of visual attention on body images. The increased accuracy and shortened reaction time in detecting a visual probe suggested an attentional bias toward body images for men with HRMD. Because the dot was displayed after the pair of images was removed, this bias must have resulted from a greater likelihood of attending to or fixating on the location previously occupied by the body image. Such findings are consistent with results from a study using word stimuli in women who are weight-dissatisfied (Gao et al., 2011), which showed that women with a negative image of their own body hastened the processing of negative body-related words and even unrelated stimuli sharing their spatial location. Our findings demonstrated a similar effect with body images for men with HRMD, confirming the maintenance of visual attention toward body images. Additional evidence of a maintained attentional bias was revealed by a longer first fixation on a body image. In the visual probe task, the increased fixation duration on body images cannot be explained by the further need of processing the image itself; rather, it must have resulted from the maintained attention at the location of the body images. Together, these findings support a bias in maintaining visual attention on images containing specific body information (Gao et al., 2011; Garner, Mogg & Bradley, 2006).

For the orientation of visual attention, both bottom-up and top-down processes can affect the speed of detecting and fixing visual stimuli (Theeuwes, 2010). Consistent with previous findings based on word stimuli (Gao et al., 2011), the higher orienting likelihood and shorter saccade latency for a body image suggest a top-down attentional bias even for visual images. It is consistent with the top-down effect of distorted self-schema on the processing of body information (Jackman et al., 1995).

The effect of internalized self-schema might be further exacerbated by the tendency of the mass media to emphasize body appearance dissatisfaction (e.g., Leit, Gray & Pope, 2002). For men with HRMD, the emphasis on body muscularity in the media would likely attract and maintain their attention on body information, leading them to perceive themselves as less muscular (Olivardia, 2001). By contrast, men with LRMD were more likely to avoid images of other men with large musculatures, which may help them maintain a healthier image of their own bodies. An established schema might have resulted from the experiences afforded by earlier education or exposure to skewed concepts of body image, contributing to further extreme behavior and negative emotion, such as shame or anxiety when exposing muscles in public. This preoccupation for individuals with HRMD would persist as the schema is further established (Pope et al., 2005).

The present study has examined, for the first time, that the attentional bias shown by populations with ED can also be found in individuals at higher risk of MD. This result offers a better understanding of the attentional processing tendency toward body-related information in high risk MD individuals; that is, they also show an attentional bias toward body-related information. This result also provides additional insight into the pathogenesis of MD. The findings of the present study also suggest that comparisons among eating disorders, body dysmorphia and MD, examining their relationships and differences, may deepen the understanding of these disorders.

To the best of our knowledge, the present study was the first to explore the cognitive characteristics of individuals at high risk for MD. Moreover, our results identifying cognitive characteristics in Asian individuals with MD in a non-English-speaking culture provide new insights into the influence of culture in the formation of MD.

In conclusion, our findings highlight a strong attentional bias for men with HRMD that is best explained by the effect of a self-schema related to negative body image. Because our results are based on a nonclinical population and the newly established CMASS questionnaire, further investigation and validation is needed to determine the cognitive mechanisms through which the attentional bias associated with mental disorders such as MD are developed.

##  Supplemental Information

10.7717/peerj.4273/supp-1 File S1 Participants recruiting procedure The procedure of recruiting participants for the present study and validity of the final samples.

10.7717/peerj.4273/supp-2 File S2 Pictures viewed The procedure of screening pictures, including evaluation of bodybuilder image using a 9-point Likert scale, and evaluation of neutral (car) image using self-assessment Manikins.

10.7717/peerj.4273/supp-3 File S3 Trial schematic Each trial began by displaying the fixation cross for 1,000 ms. This was followed by the presentation of paired images (2,000 ms) and then the visual probe (removed after a key response or 5,000 ms).

10.7717/peerj.4273/supp-4 Data S1 Raw data

## Additional Information and Declarations

### Data Availability

The following information was supplied regarding data availability: The raw data is provided as a Supplemental File.
