# Supplementary material for: Attentional biases toward body images in males at high risk of muscle dysmorphia
Source: PeerJ. 2018 Jan 16;6:e4273. doi: 10.7717/peerj.4273 (PMC5774299; doi:10.7717/peerj.4273)
Supplement: File S1 — The procedure of recruiting participants for the present study and validity of the final samples. [file peerj-06-4273-s001.pdf]

Supporting File S1

Participants Recruiting Procedure

All recruited participants worked out with free weights or machines at least three times per week. They answered a Chinese version of the Muscle Appearance Satisfaction Scale (CMASS; Jin et al., 2015) questionnaire. Their total scores on the CMASS were used to assign them into two groups at higher and lower risk of MD.

CMASS is a 17-item self-reported questionnaire, with total scores ranging from 17 to 85. Higher scores on the MASS reflect a tendency towards MD (Babusa et al., 2012; González-Martí et al., 2012; Mayville et al., 2002; Ryan & Morrison, 2010). Their total scores on the C MASS were ranked in descending order. Participants scoring in the top 27% (n = 54, total scores 51–72) comprised the HRMD group, as they were considered to be at the highest risk of developing MD, whereas participants scoring in the bottom 27% (n = 54, total scores 24–42) comprised the low risk of MD (LRMD) group (Kelley, 1939).

33 participants were recruited from each of the HRMD and LRMD groups for inclusion in the eye-tracking experiment. One participant did not finish the study, resulting in a total of 65 participants, who ranged in age from 20 to 33 years (mean, 23.66 years; SD, 2.08 years).

Sampling validity

The frequency of exercise as well as the total and subscale CMASS scores were calculated for both the HRMD and LRMD groups to assess whether the risk of MD was distinguished in the groups. Results of independent *t*-test showed significant

difference between both groups ($p < .0001$), indicating the HRMD group (top 27%) had more typical characteristics and risk of MD than did the LRMD group (see Table S1).

Table S1

Comparison with CMASS scores and frequency of excercise between the HRMD and LRMD groups.

	Group	N	Score(M±SD)	Difference in HRMD &LRMD t-statistics
Muscle Checking	HRMD	54	13.33±2.97	10.29***
	LRMD	54	7.91±2.49	
Muscle Satisfaction	HRMD	54	11.02±2.70	3.83***
	LRMD	54	9.07±2.57	
Substance Use	HRMD	54	9.13±2.42	10.43***
	LRMD	54	4.91±1.73	
Injury	HRMD	54	10.65±2.42	8.80***
	LRMD	54	6.70±2.24	
Bodybuilding Dependence	HRMD	54	13.87±2.43	12.37***
	LRMD	54	8±2.50	
Total Score	HRMD	54	58±5.47	23.00***
	LRMD	54	36.59±4.11	
Frequency of Exercise	HRMD	54	4.87±1.79	4.45***
	LRMD	54	3.43±1.57	

Note:

HRMD: Higher Risk of Muscle Dysmorphia; LRMD: Lower Risk of Muscle Dysmorphia

*** $p < .0001$.

References

Babusa B, Urbán R, Czeglédi E, Túry, F. 2012. Psychometric properties and construct validity of the Muscle Appearance Satisfaction Scale among Hungarian men. *Body Image* 9: 155-162. DOI 10.1016/j.bodyim.2011.08.005

González-Martí I, Bustos JGF, Jordán ORC, Mayville, SB. 2012. Validation of a Spanish version of the Muscle Appearance Satisfaction Scale: Escala de satisfacción muscular. *Body image,* 9: 517-523. DOI 10.1016/j.bodyim.2012.05.002

Jin X, Jin Y, Zhou S, Li X, Yang SN, Yang D, ... Yao J. 2015. The Muscle Appearance Satisfaction Scale: A factorial analysis of validity and reliability for its use on adult Chinese male weightlifters. *Body image,* 14: 94-101. DOI 10.1016/j.bodyim.2015.04.004

Kelley TL. 1939. The selection of upper and lower groups for the validation of test items. *Journal of Educational Psychology 30*: 17. DOI 10.1037/h0057123

Mayville SB, Williamson DA, White MA, Netemeyer RG, Drab DL. 2002. Development of the Muscle Appearance Satisfaction Scale A Self-Report Measure for the Assessment of Muscle Dysmorphia Symptoms. *Assessment,* 9: 351-360. DOI 10.1177/1073191102238156

Ryan TA, Morrison TG. 2010. Psychometric properties of the muscle appearance satisfaction scale among Irish and British Men. *Body Image,* 7: 246-250. DOI 10.1016/j.bodyim.2010.02.008
