# Supplementary material for: Attentional biases toward body images in males at high risk of muscle dysmorphia
Source: PeerJ. 2018 Jan 16;6:e4273. doi: 10.7717/peerj.4273 (PMC5774299; doi:10.7717/peerj.4273)
Supplement: File S2 — The procedure of screening pictures, including evaluation of bodybuilder image using a 9-point Likert scale, and evaluation of neutral (car) image using self-assessment Manikins. [file peerj-06-4273-s002.pdf]

Supporting File S2

Pictures viewed

Test images were prepared based on recommendations by Gao et al. (2011) and included images of car exteriors (neutral) and bodybuilders with large or small musculatures. Car images were taken with permission from websites, and bodybuilder images were obtained with permission from websites or captured by professional photographers employed by the authors. The body images showed seven poses used to display the muscles typically developed for bodybuilding competitions (front double biceps, front lateral spread, side chest, side triceps, rear double biceps, rear lateral spread, and abdominal and thigh poses). Each image was composed of a black background, a foreground car or muscle image (8.2cm × 9.6 cm) and a 9-point Likert scale. In addition, 5 manikins were displayed between the image and the scale to illustrate corresponding arousal and valance.

Before the eye-tracking experiment, a pilot study was conducted to assess and screen the aforementioned images. For that pilot study, 50 male undergraduate students who did not participate in the formal eye-tracking experiment were recruited. The arousal and valence of each car image was evaluated by the students using the self-assessment manikin proposed by Bradley and Lang (1994), and the musculature of each close-up image was assessed using a 9-point Likert scale. On the basis of these student assessments, the body images with the top 10 total scores were categorized as bodybuilders with large musculature for use in the formal eye-tracking experiment, and those with the bottom 10 total scores were considered bodybuilders with small musculature. The 40 car images with middle scores in perceived arousal and valence were used as neutral images in the formal eye-tracking experiment. An additional 30 images of cars with middle scores in perceived arousal and valence were

used as practice materials for the experiment.

All images were presented using the E-prime software (E-prime 2.0). Each image randomly showed only once, and was removed after the participant pressed the keyboard to indicate their evaluation outcome. The next image was displayed as soon as the key response was made.

(1) Evaluation of the musculature image using a 9-point Likert scale.

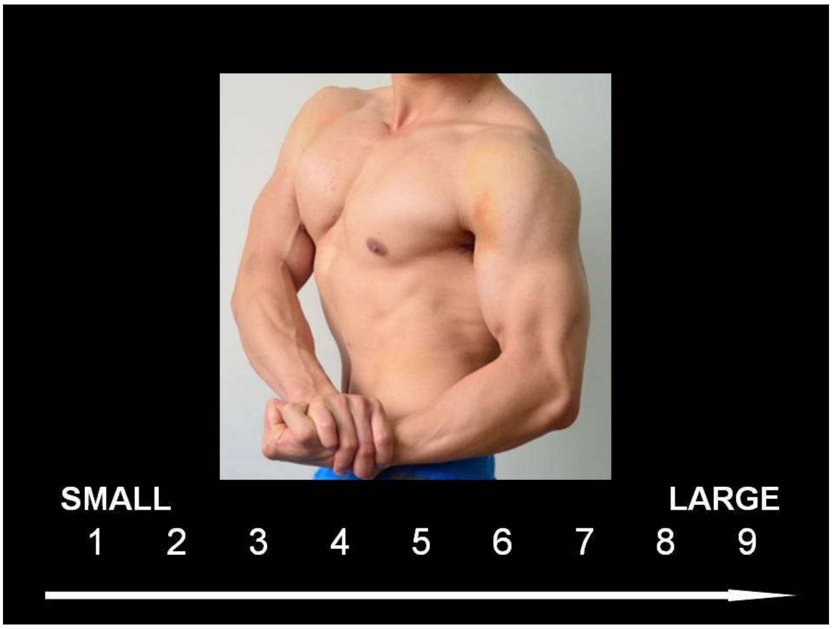

Fig.S1 Test image assessed as a large muscle. The participant used the number keys 1 to 9 on a keyboard to indicate the perceived muscle size. The larger numbers indicate more muscular images.

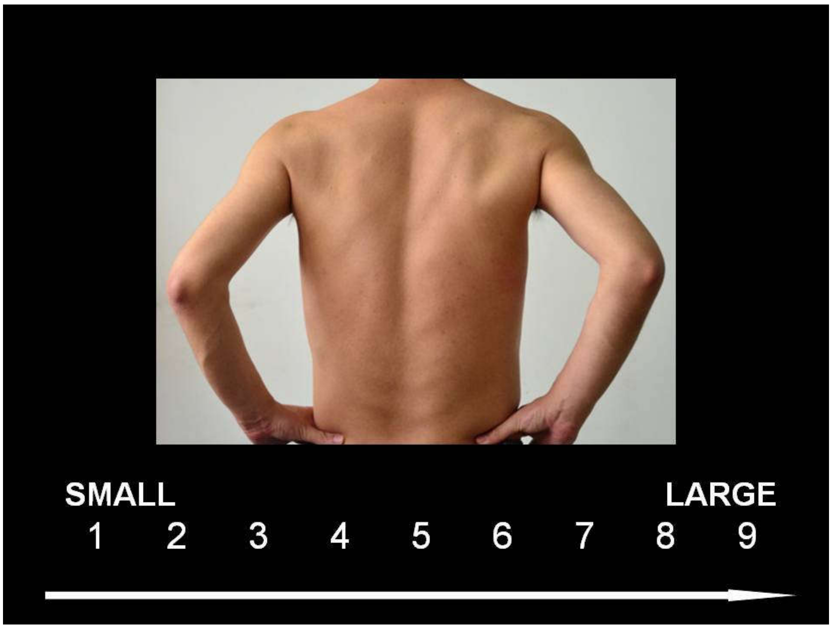

Fig.S2 Test image assessed as a small muscle size. Responses were obtained with the same method as indicated in Fig. 1

(2) Evaluation of neutral (car) image using self-assessment manikins.

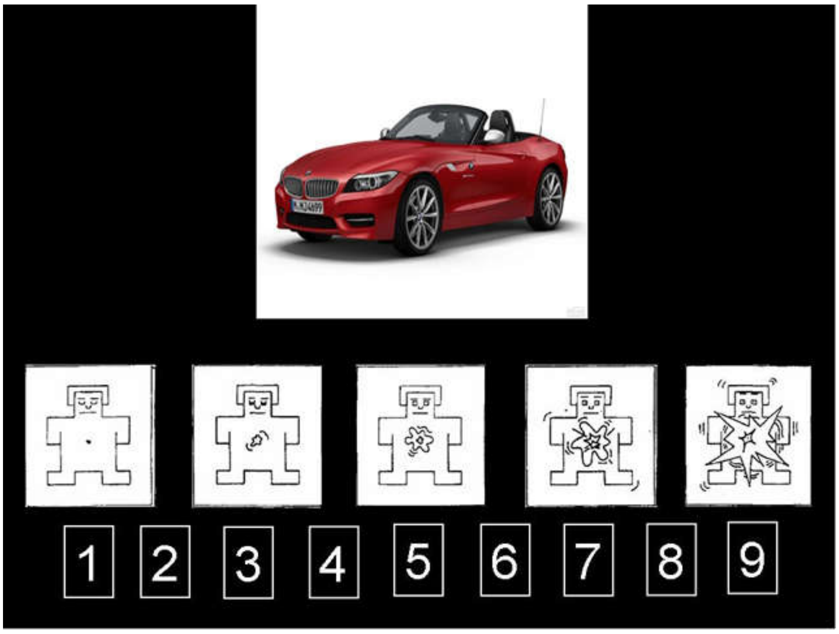

Fig.S3 Care image used to assess the level of arousal. The participant used the number keys 1 to 9 on a keyboard to indicate the perceived level of arousal as suggested by the Manikins. The larger numbers indicate greater arousal.

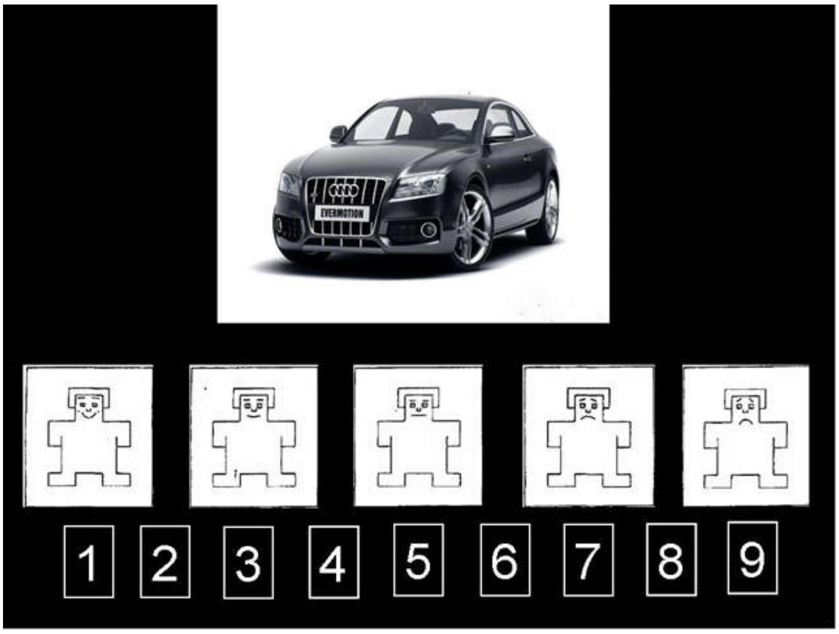

Fig.S4 Care image used to assess the level of valence. The participant used the number keys 1 to 9 on a keyboard to indicate the perceived valance. The larger numbers indicate lower valance.
