# Supplementary figures and images for: Attentional biases toward body images in males at high risk of muscle dysmorphia
Source: PeerJ. 2018 Jan 16;6:e4273. doi: 10.7717/peerj.4273 (PMC5774299; doi:10.7717/peerj.4273)

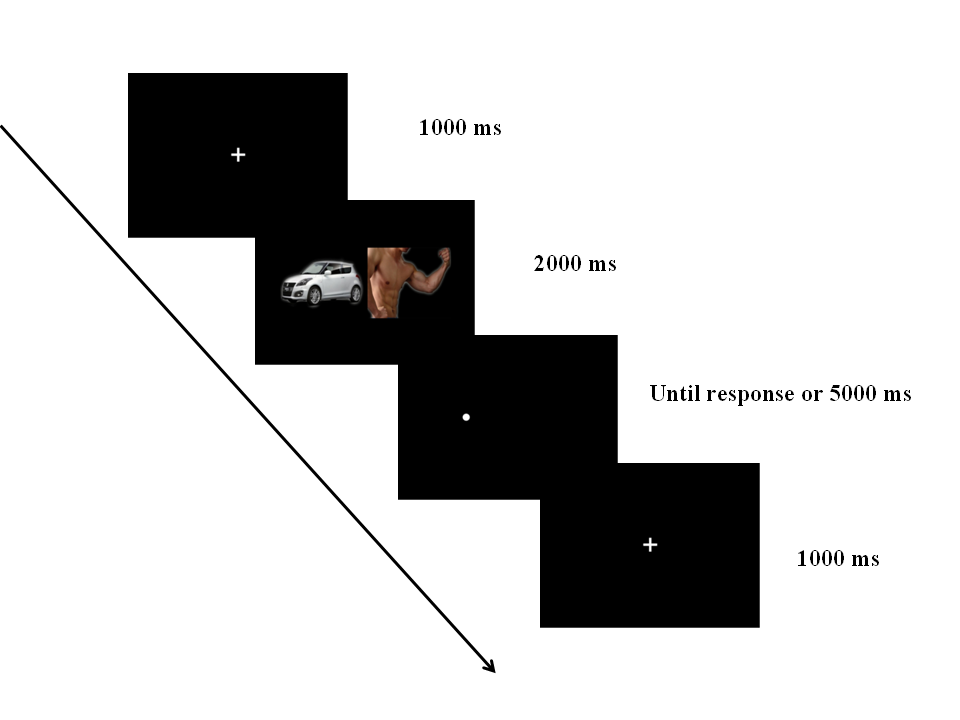

Supplement: File S3 — Each trial began by displaying the fixation cross for 1,000 ms. This was followed by the presentation of paired images (2,000 ms) and then the visual probe (removed after a key response or 5,000 ms). [file peerj-06-4273-s003.png]
